# Efficient Windows malware identification and classification scheme for plant protection information systems

**DOI:** 10.3389/fpls.2023.1123696

**Published:** 2023-02-15

**Authors:** Zhiguo Chen, Shuangshuang Xing, Xuanyu Ren

**Affiliations:** ^1^ Engineering Research Center of Digital Forensics, Ministry of Education, Nanjing University of Information Science and Technology, Nanjing, China; ^2^ School of Computer and Software, Nanjing University of Information Science and Technology, Nanjing, China

**Keywords:** protection information system, terminal protection, malware classification, image enhancement, data augmentation, deep learning

## Abstract

Due to developments in science and technology, the field of plant protection and the information industry have become increasingly integrated, which has resulted in the creation of plant protection information systems. Plant protection information systems have modernized how pest levels are monitored and improved overall control capabilities. They also provide data to support crop pest monitoring and early warnings and promote the sustainable development of plant protection networks, visualization, and digitization. However, cybercriminals use technologies such as code reuse and automation to generate malware variants, resulting in continuous attacks on plant protection information terminals. Therefore, effective identification of rapidly growing malware and its variants has become critical. Recent studies have shown that malware and its variants can be effectively identified and classified using convolutional neural networks (CNNs) to analyze the similarity between malware binary images. However, the malware images generated by such schemes have the problem of image size imbalance, which affects the accuracy of malware classification. In order to solve the above problems, this paper proposes a malware identification and classification scheme based on bicubic interpolation to improve the security of a plant protection information terminal system. We used the bicubic interpolation algorithm to reconstruct the generated malware images to solve the problem of image size imbalance. We used the Cycle-GAN model for data augmentation to balance the number of samples among malware families and build an efficient malware classification model based on CNNs to improve the malware identification and classification performance of the system. Experimental results show that the system can significantly improve malware classification efficiency. The accuracy of RGB and gray images generated by the Microsoft Malware Classification Challenge Dataset (BIG2015) can reach 99.76% and 99.62%, respectively.

## 1 Introduction

Due to increasing levels of industrialization and urbanization, dozens of major diseases and pests found on 2 billion hectares of land around the world all year round (Sun et al., 2019). The management of these diseases and pests requires a significant amount of manual input for agricultural plant protection operations, resulting in a sharp rise in labor costs (Yongliang et al., 2019; Brown et al., 2022). Therefore, intelligent plant protection information systems such as rice canopy pest monitoring systems (Li et al., 2022), field pest monitoring and forecasting systems (Liu et al., 2022), meteorological monitoring systems, and crop disease real-time monitoring and early warning systems have been widely used. The visualization and digitization of pest information improve the efficiency of pest forecasting and reduces the amount of work for plant protection staff at the grassroots level. Users can view data in real-time and manage equipment remotely through a cloud platform or mobile application to realize information management, so as to complete wireless transmission, transportation control, and information data sharing among information collection stations at all levels. However, agricultural-related data storage terminals face increasingly complex agricultural and network security situations, are threatened by various malicious software, and bear security risks such as data leakage, data theft, data loss, and data trafficking. Therefore, security systems must respond quickly to malware using new attack techniques, protect terminals from attacks, maintain the security and integrity of plant protection data, and protect the interests of farmers and the benefits of agricultural production. This paper aims to find an effective method to accurately classify malware and its variants into their families, so as to improve the malware identification and classification efficiency and enhance the comprehensive security protection capabilities of terminal systems in the construction of plant protection informatization. Many companies and scholars have proposed various malware classification techniques, which are mainly divided into two categories: signature-based classification and anomaly-based classification (Gandotra et al., 2014). Most commercial antivirus products use a signature-based approach to determine whether the software is malicious by scanning and matching signatures of known malware. This approach can quickly identify existing malware in a malware library with a low error rate but cannot identify unknown malware. Due to developments in computing power and artificial intelligence, the anomaly-based method has attracted much attention. Researchers have proposed many malware classification schemes based on this technique, which effectively overcome the limitations of signature-based methods. Malware classification schemes based on the anomaly method mainly extract features through static and dynamic analysis and selects a classification algorithm to build a model.

Dynamic analysis is the observation of the real behavior of a program at runtime which is achieved by monitoring the program’s execution in a sandbox or a virtual machine (Galal et al., 2016; Jamalpur et al., 2018). During monitoring, actions performed by programs (such as library usage, API calls (Salehi et al., 2017), network traffic, etc.) are recorded as reports. Researchers analyze characteristics in the reports to effectively categorize malware. Dynamic analysis methods attempt to discover all the actual operations of a program based on its behavior. Therefore, unknown and variant malware samples can be identified to improve the efficiency of malware classification (Ghiasi et al., 2015). However, dynamic analysis has certain limitations, such as possible infection of terminal systems, lack of suitability for real-time classification, and compromised monitoring due to evasion techniques.

Static analysis is a method of identifying and classifying executable programs without running them. It scrutinizes the “genes” of a file, rather than the current behavior which can be changed or delayed to an unexpected time in order to evade the dynamic analysis (Nissim et al., 2014). Static analysis has been proposed that mostly used by anti-malware products for automatic malware analysis. This technique allows the study of different features to build a classification system that effectively distinguishes the families to which malware belongs, such as opcode instructions (Lu, 2019), binary (Lad and Adamuthe, 2020), API (D’Angelo et al., 2020), PE header information (Rezaei et al., 2021), etc. This method can classify unknown malware and its variants, and is easier to implement than dynamic analysis. However, static malware analysis suffers from low accuracy and a high false positive rate. To overcome these shortcomings, most existing systems combine a large number of different types of features (Kim et al., 2018). Using a large number of features will cause time consumption and memory overhead, and is not suitable for real-time classification.

In recent research, static analysis methods combining malware visualization and deep learning (Liao et al., 2021; Chen et al., 2023)effectively alleviated the pressure of feature engineering technology in processing a large number of features, reduce time overhead, and make up for the shortcomings of traditional static classification methods (Yuan et al., 2020), which has achieved success in the field of malware classification. These methods visualize the binary sequence of malware as gray, RGB, or other types of images as the input of the models and use deep learning algorithms to build effective classification models that are most conducive to distinguishing the families to which malware belongs (Pinhero et al., 2021). However, in the process of visualizing the binary sequence of malware into image representation, most researchers use the zero-filling method which generates images with redundant and irrelevant features (Tekerek and Yapici, 2022). This affects the accuracy of malware classification. The existing malware benchmark datasets have the problem of unbalanced malware family data. The researchers proposed using GAN network (Park et al., 2020; Wang et al., 2022) to expand the data of small class samples to improve the efficiency of malware identification and classification. In response to the above problems, this paper proposes a malware classification system based on bicubic interpolation, Cycle-GAN, and CNNs. The accuracy of the test on the BIG2015 dataset provided by Microsoft can reach 99.76%.

The main contributions of the paper are as follows:

(i)We performed image enhancement on images converted from byte files using bicubic interpolation to preserve the integrity of malware data, addressing malware image size imbalances and image conversion distortions.

(ii)We used Cycle-GAN to perform data augmentation on gray and RGB images transformed from the BIG2015 dataset, solving the data imbalance among malware families.

(iii)We used the optimized DenseNet model to build a system to improve the efficiency of malware classification and the security capabilities of plant protection information terminal systems.

This paper is organized as follows. Section 2 provides an overview of the related work. Section 3 presents the materials and methodology. Section 4 describes the proposed system based on deep learning. Section 5 presents the experimental results and analysis. Section 6 summarizes this paper and future work.

## 2 Related work

### 2.1 Malware identification based on deep learning

Deep learning techniques such as CNN and recurrent neural networks (RNNs) have been widely used in the field of malware identification. Kumar et al (Kumar, 2021) proposed a malware classification system based on a fine-tuned convolutional neural network (MCFT-CNN). Without prior knowledge of feature engineering, binary code analysis, reverse engineering, detection, and avoidance, the system can effectively identify unknown malware samples. The classification accuracy of MalImg and BIG2015 datasets reached 99.18% and 98.63%, and the prediction time was 5.14ms and 5.15ms, respectively. The experimental results demonstrated the high efficiency of the system in identifying unknown malware, and the results on different datasets verify the universality of the system. Vasan et al. (2020) proposed an image-based malware classification system that uses a CNN architecture (IMCEC) to identify packed and unpacked malware. The experimental results show that the classification accuracy of packaged and unpackaged malware on the MalImg dataset reaches 98% and 99%, respectively. Vasan et al. (2020) proposed a malware classification system based on deep learning. The proposed fine-tuned convolutional neural network architecture (IMCFN) can effectively detect hidden code, obfuscated malware, and its variants. Experimental results show that the classification accuracy of MalImg and IoT-android datasets can reach 98.82% and 97.35%, respectively. Wang et al. (2021) proposed a gray image-based malware detection and classification system consisting of a deep efficient attention module (DEAM) and a DenseNet module. A detection accuracy of 99.3% was achieved on a dataset constructed from 1,087 benign samples collected by the authors and 1,087 malware samples randomly selected from the MalImg and BIG2015 datasets. The classification accuracy of 98.5% and 97.3% on the MalImg and BIG2015 datasets also verifies that the system can significantly improve the efficiency of malware classification. Gilbert et al (Gibert et al., 2018) proposed a deep learning system based on entropy flow to classify malware. The system used the entropy signal of wavelet transform to describe the change of entropy energy and achieved the purpose of classification by mining the similarity between the malware’s entropy streams. Experimental results show that the classification accuracy of the BIG2015 dataset reached 98.28%. Gao et al. (2020) proposed a cloud-based semi-supervised transfer learning (SSTL) framework consisting of detection, prediction, and transfer components. Experimental results on the BIG2015 dataset show that semi-supervised transfer learning can improve the accuracy of detecting components from 94.72% to 96.9%. Hemalatha et al. (2021) proposed an efficient malware classification system based on deep learning methods. The system uses a high-weight class-balanced loss function in the final classification layer of the DenseNet model, which achieves remarkable results in malware classification by addressing the data imbalance problem. The classification accuracy of the system on the MalImg, BIG2015, MaleVis, and Malicia datasets reached 98.23%, 98.46%, 98.21%, and 89.48%, respectively.

Deep learning technology can achieve more flexible malware feature representation, abstract all kinds of information contained in malware images layer by layer, and help to develop automatic and general models for identifying and classifying malware. Therefore, this paper uses the DenseNet deep learning model to build a malware classification system to effectively identify and classify malware and its variants.

### 2.2 Malware identification based on visualization technology

In the field of malware identification, researchers use visualization technology to visualize malware samples as image representations and identify malware by analyzing the visual similarity between images. Jian et al (Gao et al., 2020). proposed a deep neural network-based malware classification system (SERLA). The system utilizes image visualization and data augmentation techniques to convert the BIG2015 dataset into three-channel RGB images as input to the SERLA system. The experimental results show that the classification accuracy of the SERLA system on the BIG2015 dataset is 98.13%. Gibert et al. (2019) proposed a malware classification system based on gray images and deep learning methods. The system can capture similar characteristics between malware variants and precisely classify them into families. Experimental results show that applying the CNN model to the BIG2015 dataset achieves a classification accuracy of 97.5% and an average classification time of 0.001s. Ni et al. (2018) proposed an efficient malware classification system based on the CNN model and SimHash. The authors converted the disassembly malware code from the BIG2015 dataset into SimHash-based gray images, extracted pixel features through the CNN model, and effectively identified the family of malware. The experimental results show that the classification accuracy of the system on the BIG2015 dataset can reach 99.26%. Kalash et al. (2018) proposed a gray image-based malware classification system. The authors converted malware binary files into gray images and efficiently classified them through a CNN model. The experimental results show that the classification accuracy of the system on the Malimg and BIG2015 datasets reaches 98.52% and 99.97%, respectively. Jang et al. (2020)proposed a fastText-based local feature visualization method. This method extracts local features such as opcodes and API function names from malware and selects important local features in each malware family for embedding and visualizing through a word frequency-inverse document frequency algorithm. The experimental results show that the classification accuracy of this method on the BIG2015 dataset is about 99.65%.

The malware classification method based on visualization technology does not require disassembly and a time-consuming feature extraction process and can capture the difference between malware and its variants, so as to effectively classify malware. Therefore, malware visualization methods are beneficial to improve classification efficiency while reducing system complexity. Moreover, the visualization method can be applied to large-scale malware classification tasks without employing feature engineering techniques. This paper leverages visualization techniques to convert the BIG2015 dataset into gray and RGB image representations for the efficient classification of malware families.

### 2.3 Malware identification based on GAN networks

Generative adversarial networks (GANs), which consist of generative networks and discriminative networks, can be used for image-to-image translation and to generate high-quality images. In the field of malware classification, researchers use GANs to augment the data of classes with a small number of samples, so as to solve the problem of unbalanced malware datasets and improve classification efficiency. Tekerek et al (Tekerek and Yapici, 2022). proposed a malware classification system composed of cycle-consistent generative adversarial networks (Cycle-GAN) and DenseNet121 models. The byte files of the BIG2015 dataset were converted into gray and RGB images by B2IMG, the Cycle-GAN model was used to expand the data of the small sample family, and the DenseNet121 model was used to effectively classify the malware. The experimental results show that a classification accuracy of 99.73% is achieved on RGB images converted from the BIG2015 dataset. Rigaki et al (Rigaki and Garcia, 2018) proposed a method of generating network traffic with GANs to simulate other types of traffic. The authors modified the source code of the malware by receiving parameters from the GAN to modify the behavior of its command-and-control (C2) channel, thereby simulating Facebook chat network traffic. Experimental results show that GAN provides effective sample data for malware classification while successfully modifying malware traffic. Won et al (Won et al., 2022) proposed a generative adversarial network-based malware simulation framework (PlausMal-GAN) to augment malware image data. Experimental results show that the framework is beneficial for identifying and predicting zero-day malware-like images. Gao et al. (2022) proposed an efficient classification framework (MaliCage) for packaged malware. Experimental results show that the MaliCage framework composed of a packer detector, malware classifier, and packer GAN can classify packed malware with an accuracy of 91.66%. Singh et al. (2019) proposed a GAN-based malware image generation model (MIGAN). Experimental results show that MIGAN can improve the performance of classifiers by performing data augmentation on malware images generated from binary files, intrusion detection, and log files.

The creation of data labels for the benchmark dataset requires manual marking and is time consuming, however, GANs can learn features from real data and generate similar data without data labels. GANs can be used to generate network traffic and simulate malware data to expand the dataset, thus effectively improving the identification and classification performance. Therefore, this paper uses the Cycle-GAN model to expand the image data to balance the number of samples.

## 3 Materials and methodology

### 3.1 Dataset

The Microsoft Malware Classification Challenge Dataset (BIG2015) (Ronen et al., 2018) is a benchmark dataset in the field of malware classification. The dataset contains more than 20,000 assembly and bytecode files composed of 9 different malware families:Ramnit, Lollipop, Kelihos_ver3, Vundo, Simda, Tracur, Kelihos_ver1, Obfuscator.ACY, and Gatak. The specific data distribution is shown in **Table 1**. Each byte file contains a hexadecimal representation of the file’s binary content, excluding headers. Each ASM file contains various metadata information extracted from the binary file, such as logs of function calls, strings, etc. This paper uses the byte files in this dataset for system verification and analysis.

**Table 1:** Malware families in the training dataset.

No.	Family	Number of samples	Type
1	Ramnit	1541	Worm
2	Lollipop	2478	Adware
3	Kelihos_ver3	2942	Backdoor
4	Vundo	475	Trojan
5	Simda	42	Backdoor
6	Tracur	751	TrojanDownloader
7	Kelihos_ver1	398	Backdoor
8	Obfuscator.ACY	1,228	Any kind of obfuscated malware
9	Gatak	1,013	Backdoor
	Total	10,868	

### 3.2 Bicubic interpolation

Bicubic, Lanczos, and other bicubic interpolation algorithms have been successfully applied to data enhancement, digital splicing of multiple scenes, and information extraction (Rifman, 1973; Bernstein, 1976). Bicubic interpolation preserves the details of the original image as much as possible by interpolating or increasing the number/density of pixels in the image. In this algorithm, the value of the function *f* at the point (*x, y*) is obtained by calculating the weighted average of the nearest 16 sample points in the rectangular grid. The interpolation function in each direction is calculated using the formulas of Eq. (1) and Eq. (2).


(1) $$W(x)=\left\{\begin{array}{l} (\alpha +2){\left|x\right|}^{3}-(\alpha +3{\left|x\right|}^{2}+1\;for\;\left|x\right|\leq 1\\ \alpha {\left|x\right|}^{3}-5\alpha {\left|x\right|}^{2}+8\alpha \left|x\right|-4\alpha \;for\;1<\left|x\right|<2\\ 0otherwise \end{array}\right\}$$

Where *x* is the distance between the pixel point (*x, y*) and the last 16 sample points, *α* is usually 1 or 0.5.

For the interpolated pixel point (*x, y*) (*x, y* can be floating numbers), select a point near 4 × 4 and use Eq (2) to calculate the weighted sum.


(2) $$f\left(x,y\right)=\sum_{i=0}^{3}\sum_{j=0}^{3}f\left(x_{i},y_{j}\right)W\left(x-x_{i}\right)W\left(y-y_{i}\right)$$

**Figure 1:**
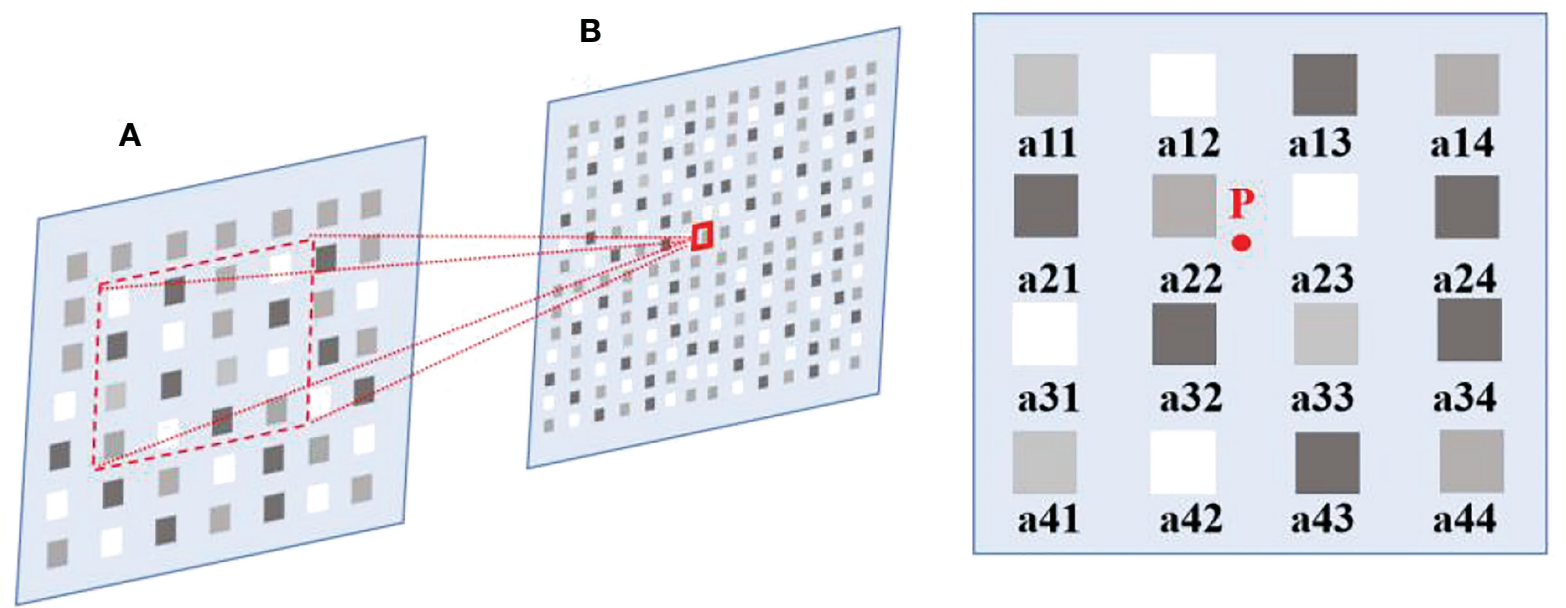
Position of the last 16 pixels of point *P*.

As shown in **Figure 1**, suppose the size of the source image *A* is *m × n*, and the size of the scaled target image *B* is *M × N*. According to the ratio, the corresponding coordinates of *B*(*X, Y*) on *A* can be obtained from *A*(*x, y*) =*A*(*X* × (*m/M*), *Y* × (*n/N*)). of the target image. Point *P* is the coordinate at (*X, Y*) corresponding to the target image *B* on the source image *A*. Assume that the coordinates of *P* are *P*(*x* + *u*, *y*+*v*, where *x*, *y* represent the integer part, and *u*, *v* represent the fractional part. As shown in **Figure 1**, the position of the nearest 16 pixels is represented by *α*(i, j=1, 2, 3, 4). According to Eq. (1), the influence factor *W* on the pixel value of point *P* is used to obtain the pixel value of the corresponding point of the target image, so as to achieve the purpose of image scaling.

In the field of malware classification, bicubic interpolation can effectively balance image size and correct distorted images (Keys, 1981). Dai et al. (2018) proposed a gray image-based malware classification system. The authors used the bicubic interpolation algorithm to equalize the size of the gray image converted from the memory dump file and used the image features extracted from the gradient histogram as input for malware classification. Experimental results show that the system achieves a classification accuracy of 95.2% on the Open Malware Benchmark dataset. Cui et al. (2018)proposed a deep learning-based malware classification system. The author used a bicubic interpolation algorithm to equalize the size of gray images converted from malicious code and used a CNN model to classify malware images. Experimental results show that the accuracy of the system on the MalImg dataset can reach 94.5%.

In this paper, the bicubic interpolation algorithm is used to enhance the gray and RGB images generated by the BIG2015 dataset to overcome the problems of pixel distortion and image size imbalance in the image conversion process. Experimental results show that image enhancement is beneficial to remove redundant and irrelevant features and improve the accuracy of malware classification.

## 4 Proposed system

**Figure 2:**
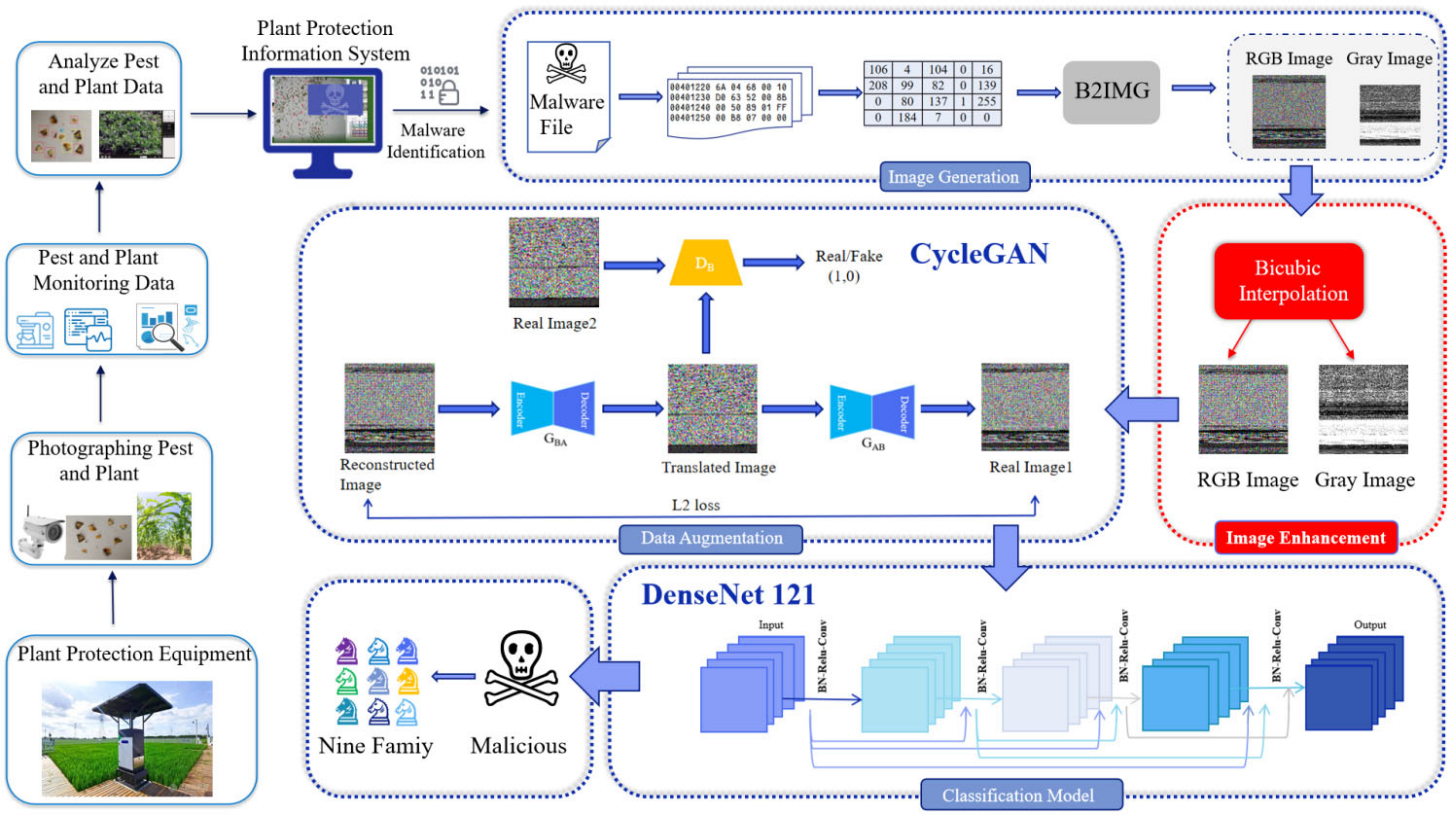
Architecture of the proposed system.

The plant protection information system is also threatened by malware when it is monitoring and defending against pests and diseases. In order to ensure the safe and stable operation of the plant protection information system, we proposed a static identification and classification system architecture of malware, as shown in **Figure 2**. The classification system utilizes bicubic interpolation, Cycle-GAN, and DenseNet121 to improve the efficiency of malware classification. The system mainly includes three parts: (1) Image generation and image enhancement, (2) Data augmentation, and (3) Classification model. Image Generation and Image Enhancement: The hexadecimal features of the byte files in the BIG2015 dataset are converted to decimal features between 0 and 255, and the malware is visualized as gray and RGB images. We use the bicubic interpolation algorithm to enhance the gray and RGB images to solve the problems of image distortion and size imbalance. Data augmentation: We use the Cycle-GAN model to perform data augmentation on a small number of samples in the BIG2015 dataset to address data imbalances among malware families. Classification model: We build an efficient malware identification and classification system using deep learning algorithms (DenseNet121).

### 4.1 Image generation and image enhancement

This paper uses the byte-to-image method (B2IMG) proposed by Tekerek et al (Tekerek and Yapici, 2022) to convert the byte files of the BIG2015 dataset into gray and RGB images, as shown in **Algorithm 1**. Firstly, the algorithm detects and removes meaningless line numbers, characters, and numbers such as “??” and “00”. Secondly, the remaining hexadecimal number is converted to a decimal value between 0 and 255 and is loaded into the pixel array. The aspect ratio of the image is obtained by dividing the total number of decimal array elements by the number of channels in the image and taking the square root of it. Finally, the decimal pixel array elements between 0 and 255 are loaded into the 2-dimensional gray image and the 3-dimensional all-0 value matrix of the RGB image to obtain the image of each malware.

Algorithm 1 Algorithm of B2IMG.
**Step 1:** While (Read Line with (filename))
**Step 2:** Line split in pixel array according to the spaces
**Step 3:** Foreach (item in pixel array)
**Step 4:** IF (item ==?? OR item<=00)
**Step 5:** Clear item
**Step 6:** ELSE
**Step 7:** Convert item hexadecimal to decimal
**Step 8:** Load the converted item in pixel array
**Step 9:** End While
**Step 10:** image size = Ceil (
$\sqrt{\frac{pixel\;array\;length}{color\;channel}}$
)
**Step 11:** Create a matrix with the size of (image size X image size X color channel)
**Step 12:** Load 0 values in matrix
**Step 13:** Reshape pixel array with (image size X image size X color channel)
**Step 14:** Load pixel array in matrix
**Step 15:** Convert matrix to image

**Figure 3:**
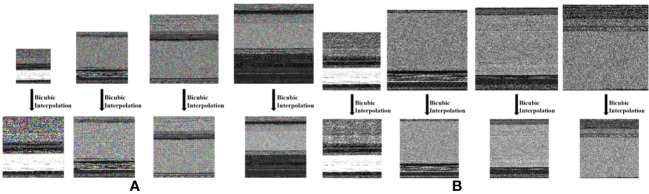
Images generated using the bicubic interpolation technique: **(A)** RGB image, **(B)** Gray image.

We used the bicubic interpolation algorithm to perform image enhancement on the RGB and gray images generated by the B2IMG method, as shown in **Figures 3A**, **B**. All image sizes are unified to 224 × 224 images as input to the DenseNet121 model.

### 4.2 Data augmentation

**Figure 4:**
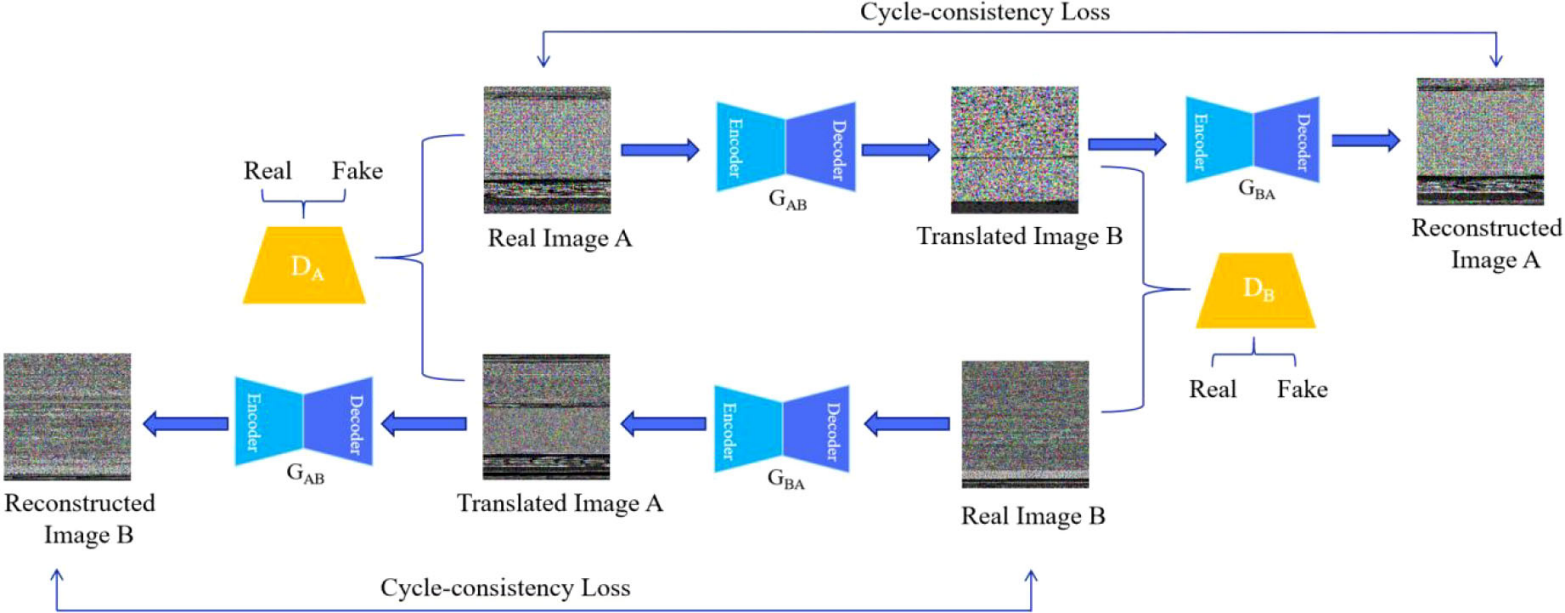
Data augmentation methods.

GANs usually require paired data, but paired data for malware images is hard to obtain in practical applications. Cycle-GAN generates image data without pairing data (Zhu et al., 2017), which greatly reduces the difficulty of malware image augmentation. Therefore, in order to solve the problem of unbalanced malware family samples, this paper uses the Cycle-GAN model to learn the features between different images of the same malware family, so as to augment the data of small sample family malware. **Figure 4** shows the malware data augmentation process, which includes 2 generative models and 2 discriminative models.

The specific process of generating the reconstructed malware image *A* from the real malware image *A* is as follows (the real malware image *B* generated the reconstructed malware image *B* is the same).

Firstly, train the generative model *G_AB_* (*G_AB_*:*A*→*B* and the discriminative model *D_B_*, obtain the adversarial loss function *L_GAN_* minimized by Eq. (3) and Eq. (4) to obtain the optimal model, and convert the real malware image *A* into the simulated malware image *B*.


(3) $${ℒ}_{GAN}\left(G_{AB},D_{B},A,B\right)=E_{a\sim P_{data}\left(a\right)}\left[{\left(D_{B}\left(G_{AB}\left(a\right)\right)-1\right)}^{2}\right]$$

(4) $${ℒ}_{GAN}\left(G_{AB},D_{B}\right)=\frac{E_{a\sim P_{data}\left(a\right)}\left[{\left(D_{B}\left(G_{AB}\left(a\right)\right)\right)}^{2}]+E_{b\sim P_{data}\left(b\right)}[{\left(D_{B}\left(b\right)-1\right)}^{2}\right]}{2}$$

denotes the collection of malware images belonging to category *A* and ${\left\{b_{j}\right\}}_{j=1}^{M}$ denotes the collection of malware images belonging to category *B*. *a*~*P*_*data*_(*a*) denotes the data distribution of malware images of category *A* and *b*~*P*_*data*_(*b*) denotes the malware images of category *B* data distribution. Secondly, the simulated malware image *B* is reconstructed into malware image *A*. By minimizing the cyclic consistency loss function *ℒ_cyc_* in Eq. (5) and identifying loss function *ℒ_idt_* in Eq. (6), the parameters of the generated model were adjusted to ensure the similarity between the reconstructed malware image *A* and the real malware image *A*.


(5) $$\begin{array}{c} {ℒ}_{cyc}\left(G_{AB},G_{BA},\lambda_{A},\lambda_{B}\right)\\ =\lambda_{A}\cdot E_{a\sim P_{data}\left(a\right)}\left[{\left|\left|G_{BA}\left(G_{AB}\left(a\right)\right)-a\right|\right|}_{1}\right]\\ +\lambda_{B}\cdot E_{b\sim P_{data}\left(b\right)}\left[{\left|\left|G_{AB}\left(G_{BA}\left(b\right)\right)-b\right|\right|}_{1}\right] \end{array}$$

(6) $$\begin{array}{c} {ℒ}_{idt}\left(G_{AB},G_{BA},\lambda_{A},\lambda_{B},\lambda_{idt}\right)\\ =E_{a\sim P_{data}\left(a\right)}\left[{\left|\left|G_{BA}\left(G_{AB}\left(a\right)\right)-a\right|\right|}_{1}\right]\cdot \lambda_{A}\cdot \lambda_{idt}\\ +E_{b\sim P_{data}\left(b\right)}\left[{\left|\left|G_{AB}\left(G_{BA}\left(b\right)\right)-b\right|\right|}_{1}\right]\cdot \lambda_{B}\cdot \lambda_{idt} \end{array}$$

The generative model can be expressed as a mapping function *G_AB_*: *A→B*, *G_BA_*: *B→A*. The discriminative model is expressed as *D_A_*, *D_B_*. Λ*_A_* and Λ*_B_* represent the cycle consistency loss weights of *A* and *B* images, respectively. Λ*_idt_* denotes the identity loss weight of the reconstructed image *A* and the real image *A*.

Finally, the optimal performance of the Cycle-GAN network is obtained by minimizing the functions *G_AB_* *, G_BA_*in Eq. (8).


(7) $$\begin{array}{l} ℒ\left(G_{AB},G_{BA},D_{A},D_{B},\lambda_{A},\lambda_{B},\lambda_{idt}\right)\\ \;={ℒ}_{GAN}\left(G_{AB},D_{B},A,B\right)+{ℒ}_{GAN}\left(G_{BA},D_{A},B,A\right)\\ \;+{ℒ}_{cyc}\left(G_{AB},G_{BA},\lambda_{A},\lambda_{B}\right)+{ℒ}_{idt}\left(G_{AB},G_{BA},\lambda_{A},\lambda_{B},\lambda_{idt}\right) \end{array}$$

(8) $$G_{AB}*,G_{BA}*=argD_{A},D_{{\mathrm{BG}}_{AB},G_{BA}}^{\max}minℒ\left(G_{AB},G_{BA},D_{A},D_{B}\lambda_{A},\lambda_{B},\lambda_{idt}\right)$$

The function ${ℒ}_{cyc}\left(G_{AB},G_{BA},D_{A},D_{B}\lambda_{A},\lambda_{B},\lambda_{idt}\right)$ in Eq. (7) represents the sum of loss functions. As shown in **Table 2**, we used the Cycle-GAN model to augment 300, 738, 400, 100, and 400 samples for the 5 families of Vundo, Simda, Tracur, Kelihos_ver1, and Obfuscator.ACY, respectively.

**Table 2:** The number of trainings, augmented trainings, and test data for BIG2015.

No.	Family	Train Data	Augmented Train Data	Test Data
1	Ramnit	1,079	0	462
2	Lollipop	1,735	0	743
3	Kelihos_ver3	2,060	0	882
4	Vundo	333	300	142
5	Simda	30	738	12
6	Tracur	526	400	225
7	Kelihos_ver1	279	100	119
8	Obfuscator.ACY	860	400	368
9	Gatak	710	0	303

### 4.3 Classification model

**Figure 5:**
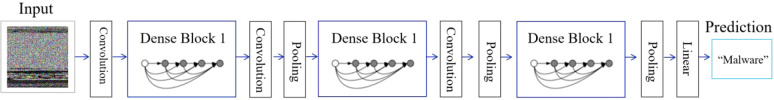
Network structure of DenseNet.

With improvements in computing power and the scale of the explosion of malware data, traditional machine-learning algorithms are no longer sufficient to identify and classify malware families effectively. The image-based deep learning method does not require specialized domain knowledge and manual parameter adjustment, and can learn independently through the model to improve classification efficiency. As shown in **Figure 5**; Huang et al. (2017) proposed a dense convolutional network (DenseNet) consisting of three dense blocks in CVPR in 2017. In each dense block module, the output features of all previous layers are used as the input of subsequent layers. The reuse of features can reduce network parameters and reduce model complexity. Compared with other networks, the DenseNet optimization problem is less difficult and can be extended to hundreds of layers. The DenseNet structure integrates identity mapping, deep supervision and attributes of different depths, which can alleviate the problem of gradient disappearance and enhance feature transfer and usage efficiency. Therefore, this paper uses the classic DenseNet (DenseNet121) to build a malware identification and classification model based on malware images. In order to prevent overfitting, we used dropout to simplify the network structure and improved the model’s generalization ability. After the FC layer of DenseNet121, we added an FC layer of size 512 to prevent overfitting and reduce redundant parameters.

## 5 Experimental results and analysis

### 5.1 Experimental setup

The BIG2015 dataset was split into two as 80% training and 20% testing. Test data was not used during the training phase. All experiments used 10-fold cross validation to prevent overfitting. According to the principle of the 10-fold CV model, 90% of the data at each fold training phase was used for training, and the remaining 10% was used in the validation phase. The final results were obtained with test data never present in the training phase. The experiment mainly uses Pytorch and the programming language is Python 3.8. Stochastic Gradient Descent (SGD) was used for optimization in this experiment, the value of learning rate was 0.03 and the value of momentum was 0.9.

This paper uses metrics such as precision, recall, accuracy and F1-score to evaluate the effectiveness of the proposed system. These metrics are widely used in the field of classification and can objectively measure the performance of malware classification systems.

Accuracy is the most commonly used measure of evaluation, and is defined as the number of samples that correctly predict the malware’s family divided by the total number of samples. Specificity represents the proportion of the sum of predicted and actual sample number not in this malware family to the sum of actual sample number not in this malware family. Precision represents the proportion of the number of samples that are correctly predicted to belong to the actual malware family to the number of samples that are predicted to belong to that malware family. Recall represents the ratio of the number of samples that are correctly predicted to belong to the family of malware to the number of families that the samples actually belong to. F1-score is a comprehensive evaluation index for measuring precision and recall


(9) $$Accuracy=\frac{TP\;+\;TN}{TP\;+\;FN\;+\;TN\;+\;FP}$$

(10) $$Specificity=\frac{TN}{TN\;+\;FP}$$

(11) $$Precision=\frac{TP}{TP\;+\;FP}$$

(12) $$Recall=\frac{TP}{TP\;+\;FN}$$

(13) $$F1=2\frac{Precision\;\times \;Recall}{Precision\;+\;Recall}$$

Where *TP* is true positive, *TN* is true negative, *FP* is false positive, and *FN* is false negative.

### 5.2 Experiment with data augmentation

In order to verify the effectiveness of the system, this paper conducts experiments on gray images and RGB images based on the original BIG2015 dataset and the augmented dataset.

#### 5.2.1 Experimental results on gray images

We used the method described in Section 4.1 to convert the malware into a gray image representation and reconstructed the generated malware gray images using the bicubic interpolation algorithm. The Cycle-GAN model was used for data augmentation to build a malware identification and classification system based on DenseNet121.

**Table 3** shows that implementing the data augmentation method on gray images can improve the AUC, specificity, precision, recall, F1-score, and classification accuracy of most malware families. The accuracy of Vundo and Obfuscator.ACY families before data augmentation were 99.66% and 99.08%. After data augmentation the accuracy increased by 0.06% and 0.28%, reaching 99.72% and 99.36%. It is worth noting that after data augmentation, the AUC and recall of the Simda family both reached 100% from 95.81% and 91.67%, respectively, indicating that all Simda family malware were accurately classified. The specificity and precision of the Tracur family were increased from 99.67% and 95.56% to 99.70% and 96.99% after data augmentation. Although the accuracy of the Kelihos_ver1 family decreased by 0.16% after data augmentation compared with that before data augmentation, the misclassification of a small data in a small sample family has little impact on the overall performance of the system. Therefore, data augmentation based on gray images can effectively improve the performance of malware classification systems.

**Table 3:** Experimental results of gray images converted from BIG2015 dataset.

		Ramnit	Lollipop	Kelihos_ver3	Vundo	Simda	Tracur	Kelihos_ver1	Obfuscator.ACY	Gatak
**Without Augmentation**	Accuracy	0.9954	0.9942	0.9985	0.9966	0.9994	0.9948	0.9991	0.9908	0.9963
	AUC	0.9901	0.9924	0.9982	0.9695	0.9581	0.9826	0.9915	0.9770	0.9905
	Specificity	0.9975	0.9956	0.9987	0.9994	0.9997	0.9967	0.9997	0.9948	0.9976
	Precision	0.9849	0.9852	0.9966	0.9859	0.9167	0.9556	0.9916	0.9592	0.9769
	Recall	0.9827	0.9892	0.9977	0.9396	0.9167	0.9685	0.9833	0.9592	0.9834
	F1-Score	0.9838	0.9872	0.9972	0.9622	0.9167	0.9620	0.9875	0.9593	0.9801
**With Augmentation**	Accuracy	0.9942	0.9926	0.9985	0.9972	0.9997	0.9942	0.9975	0.9936	0.9982
	AUC	0.9810	0.9905	0.9986	0.9759	1.0000	0.9764	0.9753	0.9939	0.9960
	Specificity	0.9996	0.9944	0.9983	0.9994	0.9997	0.9970	0.9994	0.9935	0.9987
	Precision	0.9978	0.9912	0.9955	0.9859	0.9367	0.9699	0.9832	0.9384	0.9868
	Recall	0.9624	0.9865	0.9989	0.9524	1.0000	0.9558	0.9512	0.9943	0.9934
	F1-Score	0.9798	0.9838	0.9972	0.9689	0.9565	0.9579	0.9669	0.9708	0.9901

#### 5.2.2 Experimental results on RGB images

In order to verify the generalization of the proposed system to feature images that have different textures and the effectiveness of the system, we used the method described in Section 4.1 to convert malware into RGB image representations to construct a malware identification and classification system.

As shown in **Table 4**, the use of data augmentation on RGB images can significantly improve the evaluation indicators such as AUC, specificity, precision, recall, F1-score, and classification accuracy of 8 malware families such as Ramnit, Kelihos_ver3, and Vundo. After data augmentation, the classification accuracy of Vundo, Simda, Tracur and Obfuscator.ACY families increased from 99.85%, 99.94%, 99.48%, and 99.29% to 99.91%, 99.97%, 99.60%, and 99.36%, which was an increase of 0.06%, 0.03%, 0.12%, and 0.07%. Although the classification accuracy of the Ramnit, Kelihos_ver1, and Gatak families did improve, the F1-score of the Ramnit family as a measure of precision and recall increased by 0.03%, reaching 98.18%. The Kelihos_ver1 and Gatak families still maintain high classification accuracy of 99.97% and 99.88%. Experimental results show that the augmentation of RGB image data can solve the problem of sample imbalance among malware families, which is beneficial to improve the performance of malware identification and classification systems.

**Table 4:** Experimental results of RGB images converted from BIG2015 dataset.

		Ramnit	Lollipop	Kelihos_ver3	Vundo	Simda	Tracur	Kelihos_ver1	Obfuscator.ACY	Gatak
**Without Augmentation**	Accuracy	0.9948	0.9969	0.9994	0.9985	0.9994	0.9948	0.9997	0.9929	0.9988
	AUC	0.9924	0.9961	0.9989	0.9830	0.9582	0.9730	0.9998	0.9841	0.9964
	Specificity	0.9957	0.9976	1.0000	1.0000	0.9997	0.9980	0.9997	0.9955	0.9993
	Precision	0.9740	0.9919	1.0000	1.0000	0.9167	0.9733	0.9916	0.9647	0.9934
	Recall	0.9890	0.9946	0.9977	0.9660	0.9167	0.9481	1.0000	0.9726	0.9934
	F1-Score	0.9815	0.9933	0.9989	0.9827	0.9167	0.9605	0.9958	0.9686	0.9934
**With Augmentation**	Accuracy	0.9948	0.9963	1.0000	0.9991	0.9997	0.9960	0.9997	0.9936	0.9988
	AUC	0.9855	0.9952	1.0000	0.9897	0.9615	0.9780	0.9998	0.9927	0.9978
	Specificity	0.9986	0.9972	1.0000	1.0000	1.0000	0.9990	0.9997	0.9938	0.9990
	Precision	0.9913	0.9906	1.0000	1.0000	1.0000	0.9867	0.9916	0.9511	0.9901
	Recall	0.9724	0.9933	1.0000	0.9793	0.9231	0.9569	1.0000	0.9915	0.9967
	F1-Score	0.9818	0.9919	1.0000	0.9896	0.9600	0.9716	0.9958	0.9709	0.9934

The results in **Tables 3**, **4** verify that our proposed malware identification and classification system based on bicubic interpolation, Cycle-GAN, and DenseNet121 model can effectively identify and classify malware into their corresponding families. Meanwhile, it can be seen that the classification performance of the system based on the RGB image representation of malware is better than that of the gray image representation. The classification accuracy of Lollipop, Kelihos_ver3, and Gatak families without data augmentation is 99.69%, 99.94%, and 99.88% for RGB images. These values are higher than the 99.42%, 99.85% and 99.63% accuracy for gray images. After data augmentation, the classification performance of the system based on RGB images was greatly improved in almost all families compared to gray images. Notably, the classification system based on RGB images achieved an accuracy of over 99.3% on each malware family. In particular, the classification accuracy on the Kelihos_ver3 family reached 100%. The experimental results show that the RGB image representation of malware has richer texture patterns and more feature information than the gray image representation and is more conducive to the construction of classification systems.

### 5.3 Comparison and discussion

To demonstrate the effectiveness of our proposed system, **Table 5** shows a comparison between the results of this paper and existing relevant studies based on the BIG2015 dataset. The studies used CNN, DenseNet, EfficientNetB1 and EfficientNetB7 models, as well as frameworks such as SERLA, RNN+ SSTL and CNN+ Cycle-GAN.

**Table 5:** Comparison of the proposed system to systems in the literature using the BIG2015 dataset.

Authors	Year	Models	Dataset	AUC	Precision	Recall	F1-Score	Accuracy
Gibert et al. (2018)	2018	CNN	Bytes	–	–	–	96.36%	98.28%
Gibert et al. (2019)	2020	CNN	Bytes (Grayscale)	–	94.00%	–	–	97.5%
Hemalatha et al. (2021)	2021	CNN	Bytes (Grayscale)	–	98.58%	97.84%	98.21%	98.46%
Wang et al. (2021)	2021	CNN+ DEAM	Bytes (Grayscale)	–	95.3%	95.4%	95.4%	1
Gao et al. (2020)	2020	RNN+ SSTL	Bytes+ ASM	–	96.92%	96.9%	96.81%	96.9%
Jian et al (Gao et al., 2020)	2021	SERLA	Bytes+ ASM	–	98.68%	97.93%	98.3%	98.31%
Acharya et al. (2021)	2021	EfficientNetB1	Bytes (Grayscale)	–	96.00%	97.00%	97.00%	98.57%
Pratama et al (Pratama and Sidabutar, 2022)	2022	EfficientNetB7	Bytes (Grayscale)	98.01%	97.96%	97.93%	97.93%	99.56%
Pratama et al (Pratama and Sidabutar, 2022)	2022	EfficientNetB7	Bytes (RGB)	98.30%	98.36%	98.35%	98.34%	99.63%
Tekerek et al (Tekerek and Yapici, 2022)	2022	CNN+ Cycle-GAN	Bytes (Grayscale)	98.13%	97.53%	96.50%	96.93%	99.58%
Tekerek et al (Tekerek and Yapici, 2022)	2022	CNN+ Cycle-GAN	Bytes (RGB)	98.51%	98.52%	97.16%	97.76%	99.73%
**Proposed System**	**–**	**CNN**	**Bytes (Grayscale)**	**98.33%**	**97.25%**	**96.89%**	**97.07%**	**99.61%**
**Proposed System**	**–**	**CNN**	**Bytes (RGB)**	**98.69%**	**97.84%**	**97.53%**	**97.68%**	**99.72%**
**Proposed System**	**–**	**CNN+ Cycle-GAN**	**Bytes (Grayscale)**	**98.75%**	**97.62%**	**97.72%**	**97.47%**	**99.62%**
**Proposed System**	**–**	**CNN+ Cycle-GAN**	**Bytes (RGB)**	**98.89%**	**98.90%**	**97.92%**	**98.39%**	**99.76%**

Bold text highlights authors' contributions and experimental results.

We converted byte files into image representations and adopted an improved CNN model (DenseNet121) to build the system. Compared with Gilbert et al (Gibert et al., 2018; Gibert et al., 2019). and Hemalatha et al. (2021) using the CNN model, our classification accuracy has increased by 1.44%, 2.22%, and 1.26%, reaching 99.72%.

We used the Cycle-GAN model to balance the number of samples in the malware dataset to build an identification and classification system. Compared to the classification system composed of DEAM and DenseNet (Wang et al., 2021), the accuracy, precision, and F1-Score are improved by 2.46%, 3.6%, and 2.99%, respectively. In terms of accuracy, precision, and F1-Score, our system improved by 1.45%, 0.22%, and 0.09% compared with the SERLA model (Gao et al., 2020), which was also constructed based on RGB images generated by the BIG2015 dataset, reaching 99.76%, 98.9%, and 98.39%. Compared to the above hybrid models composed of multiple classification modules, we only used a CNN model to identify and classify the families to which malware belongs, which can effectively reduce model complexity, time, and memory consumption.

We used the bicubic interpolation algorithm to enhance the malware images generated by the BIG2015 dataset to solve the problem of image size imbalance and effectively improve the performance of the malware identification and classification system. The classification accuracy of gray images and RGB images is 0.04% and 0.03% higher than that of the same image representation in (Tekerek and Yapici, 2022), reaching 99.62% and 99.76%. Compared with the EfficientNet-B model proposed by Acharya et al. (2021), the classification accuracy on gray images increased by 1.05%, reaching 99.62%. We compared our model to the B2IMG-based EfficientNetB7 model in (Pratama and Sidabutar, 2022)and achieve improved accuracy of 0.06% and 0.13% for gray and RGB images, reaching 99.62% and 99.76% respectively.

We combined image enhancement and data augmentation techniques to preserve more malware classification information while maintaining image data integrity, generating high-quality malware images for small sample families that balance malware data distribution. The 99.76% accuracy, 98.9% precision, 97.92% recall, 98.39% F1-score, and 98.89% AUC on the RGB images generated by the BIG2015 dataset prove that our proposed system can effectively identify and classify malware.

## 6 Conclusions and future work

With the exponential growth of the number of malware and its variants, the threat to plant protection information systems that store massive amounts of agricultural data is increasing. As a result, it is critical to effectively identify and classify malware. Existing malware classification schemes based on malware visualization and deep learning mainly identify and classify malware variants by analyzing the similarity of malware binary images. However, the images generated by such schemes have the problem of unbalanced image size and contain irrelevant and redundant features, which affects the accuracy of malware classification. In addition, the unbalanced data affects the classification performance of the system. Therefore, we proposed a malware identification and classification scheme based on DenseNet121 and Cycle-GAN models. The scheme used bicubic interpolation technology to enhance malware images, which solved the problem of image distortion and size imbalance caused by removing redundant and irrelevant features. Using the Cycle-GAN model for data augmentation solved the problem of unbalanced samples of malware families and effectively improves the efficiency of malware classification. The experimental results show that the AUC, precision, recall, F1-score, and accuracy of the proposed system on gray images are 98.75%, 97.62%, 97.72%, 97.47%, and 99.62%. The system can achieve 98.89%, 98.90%, 97.92%, 98.39%, and 99.76% on RGB images. Therefore, the system deployed on the plant protection information terminal can effectively prevent malware attacks, maintain the safety and integrity of plant protection data, and protect farmers’ interests and agricultural production efficiency. The BIG2015 dataset does not contain header information and cannot generate a complete image of malware. In future research, we will further collect complete malware samples for visual analysis and research, consider the problems of system complexity, cost, delay and throughput brought by system operation, balance the accuracy and time consumption, and further improve the efficiency of malware classification model.

## Data availability statement

Publicly available datasets were analyzed in this study. This data can be found here:https://www.kaggle.com/competitions/microsoft-malware-prediction/data.

## Author contributions

ZC, SX, and XR Efficient Windows Malware Identification and Classification Scheme for Plant Protection Information Systems With the exponential growth of the number of malware and its variants, the threat to plant protection information systems that store massive amounts of agricultural data is increasing. It is critical to effectively identify and classify malware. Existing malware classification schemes based on malware visualization and deep learning mainly identify and classify malware variants by analyzing the similarity of malware binary images. However, the images generated by such schemes have the problem of unbalanced image size and contain irrelevant and redundant features, which affects the accuracy of malware classification. In addition, the unbalanced data affects the classification performance of the system. Therefore, we proposed a malware identification and classification scheme based on DenseNet121 and Cycle-GAN models. The scheme used bicubic interpolation technology to enhance malware images, which solved the problem of image distortion and size imbalance caused by removing redundant and irrelevant features. Using the Cycle-GAN model for data augmentation solved the problem of unbalanced samples of malware families. The system deployed on the plant protection information terminal can prevent malware attacks, maintain the safety and integrity of plant protection data. All authors contributed to the article and approved the submitted version.
